# Cognitive and renal dysfunction in the elderly

**DOI:** 10.1590/S1980-57642013DN74000007

**Published:** 2013

**Authors:** Francisco Souza do Carmo, Sueli Luciano Pires, Milton Luiz Gorzoni, Luiz Antonio Miorin

**Affiliations:** 1 Post-Graduate Student of the Geriatric Discipline, Santa Casa de São Paulo Faculty of Medical Sciences, São Paulo SP, Brazil.; 2 Assistant Professor, Head of the Hospital Geriátrico e de Convalescentes Dom Pedro II, São Paulo SP, Brazil.; 3 Associate Professor and Head of the Geriatric Discipline, Santa Casa de São Paulo, Faculty of Medical Sciences, São Paulo SP, Brazil.; 4 Associate Professor of the Nephrology Discipline, Santa Casa de São Paulo, Faculty of Medical Sciences, São Paulo SP, Brazil.

**Keywords:** elderly, Alzheimer's disease, dementia, renal function, albuminuria

## Abstract

Cognitive impairment has been associated with several diseases and organic disturbances but few studies have explored the relationship between renal function and cognition.

**OBJECTIVE:**

The aim of this study was to compare the renal function of elderly patients with and without Alzheimer's disease, and to identify potential associated comorbidities, as well as the presence of microalbuminuria.

**METHODS:**

A group of 60 patients with dementia syndrome and probable Alzheimer's disease, and 20 patients without dementias, followed at the Geriatric outpatient unit of the Santa Casa de São Paulo Hospital, were selected for this study.

**RESULTS:**

The results showed that the groups studied differed in terms of age, gender and Mini-Mental State Exam score, but no statistical difference was found for the presence of comorbidities (diabetes mellitus, dyslipidemia and systemic arterial hypertension). A significant difference in estimated creatinine clearance was observed between the two groups, with the Alzheimer's disease patients presenting significantly lower values than control subjects. Similarly, analysis of a portion of the two groups for the presence of microalbuminuria revealed a statistically significant difference between the two groups.

**CONCLUSION:**

The study conclusions were that patients with Alzheimer's disease had lower glomerular filtration and a higher incidence of microalbuminuria, yet without having more classic risk factors for Alzheimer's disease such as systemic arterial hypertension, diabetes mellitus or dyslipidemia.

## Abstract (pt)

Alterações cognitivas são associadas a diversas doenças e distúrbios orgânicos, mas poucos estudos exploram a relação entre a função renal e a cognição.

**OBJETIVO:**

Este estudo buscou comparar a função renal de pacientes com e sem demência de Alzheimer, além de identificar possíveis comorbidades associadas e a presença de microalbuminúria.

**MÉTODOS:**

Foram selecionados 60 pacientes com síndrome demencial, provável doença de Alzheimer e 20 pacientes sem qualquer demência, que estavam em acompanhamento no ambulatório de Geriatria da Santa Casa de São Paulo.

**RESULTADOS:**

Os resultados mostraram que os grupos estudados diferiram em relação à idade, sexo e mini-exame do estado mental, mas não houve diferença estatística quanto à presença de comorbidades (diabetes mellitus, dislipidemia e hipertensão arterial sistêmica). Em relação ao clearance de creatinina observou-se uma diferença significativa entre os grupos, o com doença de Alzheimer apresentou um valor significativamente menor que o controle. Também a análise da presença de microalbuminúria entre uma parte dos dois grupos estudados, mostrou uma diferença estatística significante.

**CONCLUSÃO:**

Concluiu-se que os pacientes com doença de Alzheimer tiveram menor filtração glomerular e maior percentual de microalbuminúria, mesmo sem apresentarem mais fatores de riscos clássicos para a doença de Alzheimer, como hipertensão arterial sistêmica, diabetes mellitus e dislipidemia.

## INTRODUCTION

The ageing process, under certain circumstances, can imply evolving to potentially debilitating conditions such as dementia, one of the most prevalent neurological disorders afflicting the elderly.^1,2^

Alzheimer's disease (AD) is a progressive and fatal neurodegenerative disease characterized by cognitive and functional decline, and which sometimes causes behavioral changes. The incidence of AD rises exponentially with decades of life and, within the space of a few years, the consequent level of physical, mental and social dependence becomes critical for the majority of families of AD patients. The prevalence of the disease doubles every five years beyond 60 years of age, reaching approximately 30% at 85 years.^3^ Diabetes mellitus (DM), dyslipidemia (DLP) and systemic arterial hypertension (SAH) are considered risk factors for developing Alzheimer's disease.^4,5^

Cognitive impairment has been associated with several diseases and organic disturbances but few studies have explored the relationship between renal function and cognition. This relationship warrants attention, because it directly impacts the independence of older adults. Many studies have sought to establish the pattern of decline of glomerular filtration rate and risk factors for this dysfunction.^5-8^

Albuminuria is a marker of glomerular damage. It often occurs concomitantly with SAH and DM and is associated with older age, systolic pressure and levels of inflammatory factors. Many of these factors are present in patients with dementia. In addition, histopathological exams of kidney of individuals with albuminuria display many of the same findings identified in brain capillaries of individuals with dementia and retinal vascular disease.^8^

Given that reaching a definitive diagnosis of AD by clinical, laboratory and anatomopathological means is often challenging, efforts are warranted to confirm creatinine clearance values and the presence of microalbuminuria as markers of dementia, particularly for Alzheimer's disease.

## METHODS

**Patients.** Of a total of 702 patients (60 years or over) followed at the outpatient unit of the Geriatrics Department of the Santa Casa de Misericórdia de São Paulo Hospital, located at the Dom Pedro II Geriatrics and Convalescence Hospital in October 2008, 276 patients followed at the outpatient dementia clinic were included in this study of this group, 230 patients presented complaints of cognitive changes and were screened for cognitive decline using a test battery which included: the Mini Mental State Exam (MMSE),^9^ the Geriatric Depression Scale (GDS), the Hachinski score – a scale for diagnosing vascular dementia, the Katz index; the Lawton index, and a neuropsychological assessment.

The anamnesis, physical examination, assessment instruments listed above and laboratory exams were used to rule out other pathologies including Parkinson's-related, Lewy body, vascular, mixed and fronto-temporal dementias, progressive supranuclear palsy, delirium, depression, history of psychiatric diseases as well as potentially reversible dementias including hypothyroidism, anemia, dialytic chronic renal disease, hypovitaminosis (vitamin B12 and folic acid deficiency), neuroinfections such as neurosyphilis and neurocysticercosis, intermittent pressure hydrocephaly and intracranial expansive processes including primary and secondary neoplasias and subdural hematomas.^10^ Patients in use of renal replacement therapy malnourished, obese, edematized, amputated, or who developed terminal renal disease during the study, were excluded.

Sixty patients with dementia syndrome and probable Alzheimer's disease were Included in the present study. The diagnostic criteria for AD were based on the criteria of the National Institute of Neurological and Communicative Disorders and Stroke – Alzheimer's Disease and Related Disorders Association (NINCDS-ADRDA)^11^ and the Diagnostic and Statistical Manual of Mental Disorders (DSM IV).^12^

To serve as controls, 20 patients without AD or other types of dementia followed at the same outpatient unit were randomly included.

All patients from the control group and the respective caregivers of patients with AD, signed a free and informed consent form, previously approved by the Medical Research Ethics Committee of the Irmandade da Santa Casa de Misericórdia de São Paulo Hospital.

The diagnosis of comorbidities (DM, SAH and DLP) was based on national and international guidelines.

All patients were submitted to renal function analysis to determine creatinine clearance, serum urea concentration, and also underwent urinary sediment analysis. Clearance calculations were based on the formula by Cockcroft and Gault.^13^ Clearance values greater than or equal to 90 ml/min were considered normal, according to K/DOQI guidelines. This formula was chosen for the present study because creatinine determination measures by urine collection over a given period, such as 24 hours, offer no improvement in the estimate of glomerular filtration rate versus the values yielded by predictive formulas, and use of the formula also avoided the difficulties of bladder probe in demented patients.^14^

Two serum creatinine samples were collected from each patient with an interval of up to three months. Creatinine clearance was then calculated along with mean values. It is known that serum creatinine levels can fluctuate by up to 20% from one day to the next. Only fasting samples were taken since increases of up to 30% in sera levels may occur after food intake.^13^

Urine samples for determination of microalbuminuria were taken from 19 patients in the control group and from 18 patients in the AD group, who were selected using a random number table.^15^ The urine sample was collected in the morning with a minimum continence of two hours. The nephelometry method was employed using an N anti-serum to human albumin reagent, obtained from immunized rabbits. The equipment used was of the Siemens brand, model BN2.

Microalbuminuria in isolated samples are expressed as albuminuria/creatinuria ratio, with values of less than 30 mg albumin/gram of creatinine considered normal, whereas values between 30 and 300 mg/g indicate microalbuminuria.

**Statistical analysis.** The following formula was used to calculate sample size:

$$n=2^{*}\frac{{\left[z\left(alpha/2\right)+z\left(\mathrm{beta}\right)*SD\right]}^{\underset{─}{2}}}{{\left(mean1-mean2\right)}^{2}}$$

where: n=sample size z(alpha/2) and z(beta) obtained from the normal distribution; SD=estimated standard deviation. Mean 1 and mean 2=expected means of the two groups.

**Analysis of variables.** The quantitative variables were analyzed based on minimum, maximum values and calculations of means, standard deviations and median. Qualitative variables were assessed using absolute and relative frequencies.

Student's t-test was employed to compare means of the two groups, and Mann-Whitney's non-parametric test was used for non-normal data.

Homogeneity among proportions was evaluated using the Chi-squared test, or Fisher's exact test when expected frequencies were less than five.

Means and gender of the two groups were compared using two-way variance analysis.^16^

All statistical analyses were carried out using Windows Office 2000 and the statistical software package SPSS 15.0 for Windows.

The level of significance used for all tests was set at 5%.

## RESULTS

Patients from the AD group were significantly older (79.5 y) than subjects from the control group (73.3 y) (p<0.001) and had significantly lower MMSE scores (p<0.001).

The mean age of study patients was 79.5 years (ranging from 60 to 89 years). Regarding gender distribution, 14 patients were men and 46 women. The mean score of AD patients on the MMSE was 14.78. Mean age of controls was 73.35 years (ranging from 66 to 87 years). Regarding gender distribution, 10 control patients were men and 10 women. The mean score of controls on the MMSE was 27.1 (Table 1).

**Table 1:** Mean values, standard deviation, median, minimum and maximum for age and Mini Mental State Exam score (MMSE).

	Group	S	Mean	SD	Median	Minimum	Maximum	p*
Age	Control	20	73.35	5.13	73	66	87	<0.001
	AD	60	79.50	6.27	80	60	89	
MMSE	Control	20	27.10	2.65	27.50	20	30	<0.001
	AD	60	14.78	6.81	15.50	0	29	

Age(*) Student's t-test; MMSE (*) Mann-Whitney test; S: sample size, SD: standard deviation.

The groups also differed in terms of gender distribution, where the AD patient group contained more women than the control group (p<0.024). However, no difference was found between the groups for the risk factors SAH (p=0.720), DM (p=0.185) or DLP (p=0.302) (Table 2).

**Table 2:** Absolute and relative frequencies of Gender and of Risk factor.

		Group					p
		Control (n=20)			AD (n=60)		
		n	%		n	%	
Gender	Women	10	50.0		46	76.7	0.024**
	Men	10	50.0		14	23.3	
Risk factor	SAH	18	90.0		50	83.3	0.720*
	DM	6	30.0		9	15.0	0.185*
	DLP	12	60.0		28	46.7	0.302**

* Fisher's exact test;

** Chi-Square test.

With regard to creatinine clearance estimated according to age, weight and gender, a significant difference was observed between the groups, with the AD group having a significantly lower value than the control group (p<0.001) (Table 3A).

**Table 3A:** Mean values, standard deviation, median, minimum and maximum estimated creatinine clearance.

Group	S	Mean	SD	Median	Minimum	Maximum	p*
Control	20	68.95	18.42	66.29	43.37	102.63	<0.001
AD	60	51.66	18.56	48.09	22.36	117.85	

* test t Student; S: sample size; SD: standard deviation.

Analysis of the effect of gender and group factors showed a significant interaction between the two factors for creatinine clearance value (p=0.044). Although no significant difference was found in the control group (p=0.538), a significantly higher value was found for male gender in the AD group (p<0.001). A significantly higher value for women was found in the control group compared to the AD group (p<0.001) (Table 3B).

**Table 3B:** Mean values, standard deviation, median, minimum and maximum estimated creatinine clearance.

Group	Gender	S	Mean	SD	Median	Minimum	Maximum
Control	Women	10	66.65	17.04	64.02	44.59	95.79
	Men	10	71.25	20.34	71.72	43.37	102.63
AD	Women	46	46.30	12.95	46.11	22.36	75.34
	Men	14	69.30	23.38	71.97	30.85	117.85

S: sample size; SD: standard deviation.

Subsequently, groups were analyzed for a partition value of 60 mL/min indicative of moderate renal failure according to stage 3 of KDOQI guidelines. A significant difference between the two groups was found, revealing a higher percentage of individuals with chronic renal disease in the dementia group (p<0.007) (Table 4).

**Table 4:** Absolute and relative frequencies of estimated creatinine clearance

Clearance	Group					p*
	Control (n=20)			AD (n=60)		
	n	%		n	%	
< 60	8	40.0		44	73.3	0.007
≥60	12	60.0		16	26.7	

* Chi-Square test.

Analysis of the presence of microalbuminuria in a portion of the two groups revealed a statistically significant difference between the two, where the AD subgroup was found to have a greater incidence of microalbuminuria (p=0.02) (Table 5).

**Table 5:** Absolute and relative frequencies of microalbuminuria.

Risk factor	Group					p
	Control (n=19)			AD (n=18)		
	s	%		s	%	
Microalbuminuria	0	0.0		5	27.7	0.02*

* Fishers exact test.

## DISCUSSION

This study showed that the presence of chronic renal disease was highly frequent among AD patients followed at our institution. Scant data is available in the literature assessing the prevalence of chronic renal disease (CRD) in this patient group.^5-8^ This finding has sufficient relevance to justify renal function assessment in all elderly patients with cognitive changes.^17^

AD is known to be the most prevalent form of neurodegenerative dementia in the elderly population. In 2000 there were 4,500,000 cases of AD in the USA and this figure is set to rise to 14,000,000 by 2050.3 Global projections for 2050 stand at 102,000,000 AD patients, 40% of whom will have reached an advanced stage of the disease.^18^ According to an anatomopathological study published in 2002, AD was also the most common condition among patients from a Florida brain bank, accounting for 77% of the 382 brains assessed, while 77% of vascular dementia cases were found to have anatomopathological changes consistent with Alzheimer's disease.^19^

Clinical diagnosis of dementia remains questionable in terms of accuracy since there is not always diagnostic concordance among criteria employed. The NINCDS-ADRDA criteria, adopted in most studies to determine a clinical diagnosis of Alzheimer's disease, are 62.5% to 93.0% consistent with pathological anatomy. Pathological anatomy is regarded as the "gold standard" but there is no consensus on the criteria for definitive diagnosis of Alzheimer's disease.^19^

In line with the findings of other authors, the present study identified a higher prevalence of female AD patients. This may be explained by the higher life expectancy of women creating a bias in the proportion of women in the elderly population, associated with longer survival of AD women compared to men.^20^

In the present study, AD patients were found to be older (79.5 y) than controls (73.35 y), a difference reaching statistical significance (p<0.001). This may be partially explained by the fact that studies indicate that age is the only proven risk factor for developing AD, i.e. the older the individual, the greater the incidence and prevalence of the disease.^21^

With regard to estimated creatinine clearance evaluated according to age, weight and gender, a significant difference was observed between the groups, with the AD group having a significantly lower clearance value than the control group (p<0.001). This may be explained by the older overall age of the group, yet no association between low clearance and the risk factors studied (SAH, DLP and DM) was evident in these patients.

Another study found the factors DM, SAH, heart disease and smoking to be associated with higher risk for AD. The risk increased 3.4-fold in individuals presenting three or more of these factors. Therefore, the risk of AD increases with number of vascular risk factors.^4^

Recent data published by Algotsson and Winbland^22^ indicated a concomitant relationship between glomerular dysfunction and blood-brain barrier integrity in AD patients. The study showed these patients had compromised barriers due to deposition of beta-amyloid peptides in the cerebral arterioles, responsible for the forming of senile plaques. Renal function may have a significant impact on blood-brain barrier function given renal dysfunction may reduce the elimination of this protein leading to renal and cerebral amyloid angiopathy.

The question remains as to whether CRD, i.e. individuals with clearance lower than 60 mL/min, can be considered a risk factor for cognitive change, in the light of studies demonstrating lower MMSE scores in individuals with impaired renal function.^8,17,23^

In the present study, a significant difference between the groups was found regarding the presence of microalbuminuria. An important study published by Barzilay et al [8] found a strong association among albuminuria, chronic renal disease and dementia. These authors postulated that albuminuria increases the likelihood of an individual evolving with dementia and that this could be treatable. They also emphasized that the albuminuria could be a complication of highly prevalent diseases in the elderly population such as DM and SAH.^24^

We believe the most salient aspect is the fact that AD patients presented a higher incidence of microalbuminuria coupled with lower creatinine clearance, and therefore had relative hyperfiltration of remnant nephrons.

Microalbuminuria is a known marker of absolute hyperfiltration in diseases such as DM and in situations of renal parenchymal loss, for instance following ablations. It is also a marker of early renal damage in SAH in which nephron loss progressively increases the burden on remnant nephrons. Our findings point to a possible association between function loss and change in the ability of the kidney to reabsorb the albumin filtered, detected as microalbuminuria in this study. Our group presented a higher proportion of patients with AD who had microalbuminuria. This may be suggestive of a new early marker of AD.

The results of this study allow us to conclude that patients with Alzheimer's disease have lower glomerular filtration and a higher incidence of microalbuminuria, yet without having more classic risk factors for Alzheimer's disease such as systemic arterial hypertension, diabetes mellitus and dyslipidemia. Finally, further studies are needed involving a greater number of patients in order to confirm our preliminary hypothesis.
